# Differential Privacy Preserving Voice Conversion for Audio Health Data

**DOI:** 10.1002/alz.091280

**Published:** 2025-01-09

**Authors:** Nauman Dawalatabad, Meysam Ahangaran, Cody Karjadi, Lampros Kourtis, Philip Joung, Vijaya B. Kolachalama, Rhoda Au, James Glass

**Affiliations:** ^1^ Massachusetts Institute of Technology, Cambridge, MA USA; ^2^ Boston University Chobanian & Avedisian School of Medicine, Boston, MA USA; ^3^ ramingham Heart Study, Boston University Chobanian & Avedisian School of Medicine, Boston, MA USA; ^4^ Clinical and Translational Science Institute, Tufts University, Boston, MA USA; ^5^ Gates Ventures, Seattle, WA USA; ^6^ Boston University School of Public Health, Boston, MA USA; ^7^ Framingham Heart Study, Boston University Chobanian & Avedisian School of Medicine, Boston, MA USA

## Abstract

**Background:**

Speech is a predominant mode of human communication. Speech digital recordings are inexpensive to record and contain rich health related information. Deep learning, a key method, excels in detecting intricate patterns, however, it requires substantial training data. Laboratories have invested significantly to gather extensive digital voice datasets for health insights. The challenge lies in securely sharing this data while protecting the speaker's privacy.

**Method:**

We applied a Generative Adversarial Network (GAN) approach. GANs can generate a voice that closely resembles a real voice and is composed of four key components: (i) Generator, (ii) Formant Extractor, (iii) Speaker Embedding Extractor, and (iv) Discriminator. Model training involves leveraging adversarial loss, wherein the generator strives to produce a voice that convincingly mimics reality to deceive the discriminator. Simultaneously, the discriminator endeavors to discern whether the generated voice is authentic or synthetic. Eventually the generator produces a voice that resembles closely to the real voice.

**Result:**

We performed zero‐shot voice conversion using an emotion preserving GAN. The model preserves the fundamental frequency trajectories. This is one of the most important features in dementia classification.

**Conclusion:**

By successfully altering voice prints while preserving sound quality, our initial findings suggest a way for sharing raw digital voice recordings. Future efforts will extend beyond preserving intonation patterns, focusing on preserving additional dementia related markers in the original signal.

